# TB or not to be: what specificities and impact do antibodies have during tuberculosis?

**DOI:** 10.1093/oxfimm/iqab015

**Published:** 2021-07-15

**Authors:** Clemens Hermann, Carolyn G King

**Affiliations:** Department of Biomedicine, University of Basel, University Hospital of Basel, CH-4031 Basel, Switzerland

**Keywords:** antibodies, B cells, tuberculosis, BCG

## Abstract

Tuberculosis, an infectious disease caused by *Mycobacterium tuberculosis* (Mtb), is a major cause of global morbidity and mortality. The primary barrier to the development of an effective tuberculosis vaccine is our failure to fully understand the fundamental characteristics of a protective immune response. There is an increasing evidence that mobilization of antibody and B cell responses during natural Mtb infection and vaccination play a role in host protection. Several studies have assessed the levels of Mtb-specific antibodies induced during active disease as well as the potential of monoclonal antibodies to modulate bacterial growth *in vitro* and *in vivo*. A major limitation of these studies, however, is that the specific antigens capable of eliciting humoral responses are largely unknown. As a result, information about antibody dynamics and function, which might fundamentally transform our understanding of host Mtb immunity, is missing. Importantly, Mtb infection also induces the recruitment, accumulation and colocalization of B and T cells in the lung, which are positively correlated with protection in humans and animal models of disease. These ectopic lymphoid tissues generally support local germinal center reactions for the proliferation and ongoing selection of effector and memory B cells in the mucosa. Efforts to leverage such responses for human health, however, require a more complete understanding of how antibodies and B cells contribute to the local and systemic host Mtb immunity.

Despite major efforts to control the ongoing epidemic, tuberculosis (TB) remains the leading cause of death from a single infectious agent worldwide. In 2018, the World Health Organization reported 10 million new cases of active TB and 1.45 million deaths [1]. It is further estimated that 2 billion people currently maintain a latent infection with *Mycobacterium tuberculosis* (Mtb), the causative agent of TB, and are therefore at risk of developing active disease at some point during their lives. Even though antibiotics exist to treat active disease, multidrug-resistant strains of Mtb have spread, with 3.4% of new TB cases and 18% of previously treated cases showing resistance. These numbers suggest that chemotherapy alone will not be sufficient to control the ongoing TB epidemic [1]. One solution could be the design of effective vaccines to prevent infection or development of active disease. However, the only licensed vaccine to date is *M.bovis* Bacillus Calmette–Guérin (BCG), which has shown variable efficacy in different populations [2].

In order to protect against infections with pathogens, the immune system generally employs three strategies: it can control and minimize the pathology of an ongoing infection; sterilize an existing infection; or develop resistance to future infections by the same pathogen. Controlling TB disease is clearly of utmost importance given the large number of people who are latently infected. Sterilization of an existing infection largely relies on cell-mediated immunity that involves the killing either internalized microbes or infected cells. This arm of the immune system has been investigated during natural infection with Mtb in detail [3–5], and although clearly important, is not necessarily sufficient to expunge the bacteria. Lastly, prevention of many infections can be achieved by the induction of a protective antibody response, which is exactly how most prophylactic vaccines work [6]. It is therefore reasonable to consider what role antibodies could play in protection against infection with Mtb or the development of active disease. In general, antibodies might play a role in preventing Mtb infection after initial exposure to inhaled bacteria, but also when a latently infected person progresses to active disease leading to dissemination of bacteria into the extracellular space. As the infectious inoculum of Mtb is typically very small (1–36 bacilli in the case of primary exposure), an effective humoral response in the lung could be the difference between infection and resistance [7]. In contrast to cell-mediated immunity, however, very few studies have investigated the impact of antibodies and B cells during Mtb infection in humans, and even less is known about humoral responses in the lung mucosa, the site of infection and primary disease. As for many infectious diseases, animal models have been used to assess antibody-mediated protection *in vivo*. However, many of these studies have yielded inconsistent results, potentially attributable to (i) the use of mice with different genetic backgrounds—some of which are more or less susceptible to Mtb; (ii) infection with distinct Mtb strains whose virulence varies; (iii) different routes of Mtb administration; and (iv) varying Mtb doses grown either in the presence or absence of detergent. Further complicating matters, the widespread use of purified protein derivative (PPD) to measure the level of antibody responses might seriously limit the breadth of specificity that can be considered. PPD is extracted by autoclaving Mtb at high temperatures, which denatures all proteins and removes the 3D structure of any conformational epitopes. Moreover, it has been shown that the proteome of PPD is dominated by only 6% of the Mtb proteome [8]; thus, excluding a large number of potentially valuable antibody targets. Despite these limitations, several more recent studies on antibodies elicited during natural infection in humans have raised hopes that antibody responses could indeed play a supporting role in the fight against TB (see also [9, 10]).

## MONOCLONAL ANTIBODY STUDIES DURING MTB INFECTION IN MICE

In contrast to other infectious respiratory diseases, such as influenza and coronavirus disease 2019 (COVID-19), for which the antigenic targets of protective antibodies are well defined [11, 12], the predominant antibody specificities during Mtb infection are largely unknown. Although the Mtb genome encodes over 4000 proteins most studies have examined only a limited subset of these proteins. What we know about these antigens stems primarily from a set of Mtb challenge studies in mice that tested the protective effect of monoclonal antibodies targeting three well-known Mtb antigens: heparin-binding hemagglutinin (HBHA), alpha-crystallin and arabinomannan (AM), the sugar component of the glycolipid lipoarabinomannan (LAM). Here, monoclonal antibodies reduced bacterial burden, enhanced containment of bacteria or reduced lung pathology [13–19]. What is the function of these antigens during infection? HBHA is a surface-exposed protein that interacts with proteoglycans and can facilitate Mtb entry into epithelial cells *in vitro* [14, 20]. During infection, HBHA was shown to be required for extrapulmonary dissemination, as mucosal administration of Mtb lacking HBHA expression impaired its ability to spread to other organs, such as the spleen, in mice [14]. Similar to HBHA, LAM is found in the bacterial cell envelope and constitutes a major component of the cell wall. During infection, LAM interacts with mannose receptor on host cells, thereby promoting bacterial uptake [21]. Alpha-crystallin (also called 16-kDa antigen or HspX) is a cytosolic protein that has also been detected in the cell envelope of Mtb [22]. Alpha-crystallin is expressed at relatively low levels during exponential bacterial growth but highly abundant during stationary phase [23]. Accordingly, alpha-crystallin is essential for bacterial survival during periods of disease latency when Mtb also undergoes metabolic adaptation to survive under conditions of oxygen deprivation, nutrient depletion and low pH [24, 25]. Although in sum, these studies clearly demonstrate a protective role for Mtb-targeted monoclonal antibodies, the isolation and testing of naturally derived protective antibodies have been largely ad hoc. What other antigens are targeted by humoral immunity during the course of human Mtb infection?

## ANTIGENS TARGETED BY HUMORAL IMMUNITY DURING MTB INFECTION IN HUMANS

Early serological studies in humans showed that active TB disease provokes a strong antibody response. It was soon recognized that some of the Mtb antigens targeted by antibodies during active disease might facilitate early diagnosis, particularly in resource-limited settings. In the pregenomics era, identification of antigens relied on testing of protein fractions and secreted proteins from *in vitro* cultured Mtb for antibodies from Mtb infected animals as well as infected TB patients [26]. Thus, proteins in the culture filtrate of Mtb replicating in liquid media were subsequently found to act as strong B cell antigens, especially in patients with active disease [27, 28]. One caveat of this discovery approach, however, is that bacteria grown in the presence of mild detergent might lead to partial extraction of proteins from the cell envelope and artificially high abundance in the culture filtrate. Nevertheless, the most frequently identified antigens for serological studies include antigen 85 (Ag85), PstS1, LpqH, MPT32 and malate synthase G, in addition to the already mentioned targets alpha-crystallin, HBHA and AM/LAM [29–31]. Most of these antigens are detected in the cell envelope and are at least partly involved in bacterial interaction with macrophages. The Ag85 complex, consisting of the subunits Ag85A, Ag85B and Ag85C, is a secreted protein that also exhibits cell wall mycolyltransferase activity and is required for the biosynthesis of cord factor, a virulent glycolipid that drives granuloma formation [32]. Ag85 has a high affinity for fibronectin and facilitates attachment of Mtb to murine alveolar macrophages. PstS1 is a surface-exposed lipoprotein involved in the uptake of inorganic phosphate, an essential but often limiting nutrient in the microenvironment. Like Ag85, PstS1 can also act as an adhesin for binding to human and mouse macrophages [33]. LpqH, another cell surface-exposed antigen, is a glycoprotein that acts as a TLR2 agonist, inducing upregulation of MHC II and cytokine secretion by macrophages [34, 35]. The antigen MPT32 is secreted by Mtb early on during the course of disease progression but has also been detected in the cell envelope fraction of Mtb. MPT32 functions as an adhesin and is suggested to be involved in the invasion of epithelial cells [36]. Lastly, malate synthase G is a cytosolic protein that functions in glycolate metabolism and elicits strong antibody responses during active TB [37].

Most of these antigens are at least partly located in the cell envelope of Mtb, highlighting the importance of this layer in generating antibody responses during infection. In general, antibodies that bind to cell-surface exposed antigens can lead to opsonization, thereby impacting bacterial uptake and intracellular trafficking by phagocytic cells [6, 38]. Accordingly, bulk serum IgG antibodies from Mtb exposed but uninfected healthcare workers were shown to contain a fraction of surface-specific antibodies that inhibit Mtb growth *in vitro* and reduce bacterial burden in a mouse challenge model [39]. Although depletion of antibodies binding to the cell surface of intact Mtb led to loss of this protective effect, efforts to identify the Mtb antigens using protein microarrays were unsuccessful. Apart from polysaccharides and proteins, the mycobacterial cell envelope also contains a variety of glycolipids that are strong inducers of nonconventional T-cell responses [40]. Could some of these glycolipids also be targeted by antibody responses during infection? To date, only IgG antibodies recognizing cord factor (trehalose 6,6′-dimycolate) have been reported [41], although there has not yet been a study addressing this question thoroughly. Importantly, and in line with the idea that antigens in the capsular Mtb fraction contain valuable antibody targets, mycobacteria cultured in the absence of detergents that otherwise strip the mycobacterial capsule elicit superior antibody responses in mice [42].

## ATTEMPTS TO LINK ANTIBODY SPECIFICITY AND TB DISEASE

Although the Mtb antigens discussed above are useful for measuring antibody titers, only a few studies have assessed the functional impact of humoral Mtb immunity by assessing the relationship between antibody specificity and disease state. One important consideration is the classification of TB disease, particularly as exposure to Mtb does not necessarily lead to successful infection but may still induce immune reactivity. Mtb infection is clinically assessed by two tests: the tuberculin skin test (TST) and the interferon-γ (IFN-γ) release assay (IGRA). The TST involves injection of PPD into the skin, which results in a delayed-type hypersensitivity reaction in individuals with previous Mtb exposure or infection. IGRA measures IFN-γ production by Mtb-specific T cells following *ex vivo* stimulation with Mtb peptides, ESAT6 and CFP10. Individuals who remain TST− and IGRA− despite high exposure to people with clinically diagnosed Mtb have been termed ‘resisters’ or ‘long-term controllers’ [43]. Notably, a recent study demonstrated significant IgM, IgG and IgA antibody titers in a cohort of Ugandan resisters who were highly exposed to Mtb through household contacts. Antibodies from the serum of these individuals were reactive to PPD, Ag85, ESAT6/CFP10, alpha-crystallin, GroES and LAM. Although the specificities of antibodies isolated from resisters and individuals with latent TB infection were largely overlapping, bulk PPD-specific antibody responses in resisters were qualitatively distinct from individuals with latent TB, with higher avidity IgG and higher titers of antibodies capable of eliciting IFN-γ secretion of NK cells (the function of antibodies to individual Mtb antigens was not determined) [44]. Antibody efficacy has also been reported to diverge according to distinct glycosylation profiles observed between cohorts of active and latent TB [45, 46]. Although glycosylation of the Fc antibody domain clearly regulates IgG structure and effector function, the link between glycosylation and function reported in these studies might also reflect changes in antibody specificity. For example, increased agalactosylation that correlates with reduced antibody efficacy during active TB could represent the expansion of nonspecific plasma cells, which poorly target Mtb.

A more refined linking of antibody specificity and disease was performed by Wardemann *et al*. By isolating plasmablasts from active TB patients as well as Mtb-exposed healthcare workers, the authors identified a number of antibodies specific for the surface antigens LAM and HBHA [47]. Mtb-specific plasmablasts isolated from healthcare workers showed a high frequency of IgA+ B cells suggesting their mucosal origin. Importantly, IgA antibodies were able to prevent epithelial cell infection *in vitro* while IgG antibodies with the identical specificity either promoted infection or had no effect. These findings were also recapitulated in polyclonal IgA and IgG antibodies isolated from the serum underscoring the opposing and isotype-dependent impact of these antibodies. A similar study by Freund *et al.* generated a panel of monoclonal antibodies specific for PstS1, using memory B cells isolated during active TB disease [48]. Despite the more inflammatory environment-associated active TB, two antibody clones showed protective effects in a whole blood Mtb growth inhibition assay. Consistent with these observations, transfer of anti-PstS1 monoclonal antibodies several hours prior to challenge with ∼100 CFU of aerosolized Mtb, a relatively high infectious inoculum compared with ‘natural’ transmission, resulted in decreased bacterial burden in the lung. These findings clearly indicate induction of protective antibodies, even in the case of active TB disease. In a macaque model of Mtb infection, high abundance of IgG and IgA plasma cells in the lung correlated with high bacterial load [49]. Taken together, these observations raise the question of whether such antibodies, if protective, arise too late to prevent the onset of active disease.

A further report, looking at a broader spectrum of human Mtb infection, characterized the serum IgG response to AM, the delipidated form of LAM which is a major component of the capsular layer of Mtb [50]. In this study, healthy TST− individuals were compared with two patient groups with latent TB infection (TST+ and IGRA− or IGRA+) and patients with active TB disease. IgG antibodies targeting AM in latently infected patients were found to enhance uptake of Mtb and intracellular killing by human macrophages *in vitro*. This effect was dependent on anti-AM-specific antibodies because depletion of AM-specific antibodies abrogated the effect [51]. Most importantly, this protective response was confirmed using an *in vivo* mouse challenge model in which polyclonal AM-antibodies purified from the serum of selected individuals with latent TB infection were transferred into mice prior to low dose Mtb challenge. In this study, three out of four patient antibodies were capable of lowering the bacterial burden although the magnitude of this effect was relatively small [51].

## THE SEARCH FOR NOVEL ANTIGENS USING MTB PROTEIN MICROARRAYS

Given how little we know about antibody specificity, unbiased methods to identify relevant targets are absolutely essential to assess the dynamics and functional impact of humoral immunity. Protein microarrays are one such tool for identifying antibody reactivity across a wide array of Mtb antigens. The first reported Mtb protein microarray used *Escherichiacoli* as an expression system and spotted proteins without further purification onto nitrocellulose slides. By comparing antibodies from patients across a wide spectrum of disease, the authors were able to identify a subset of Mtb targets that associated with active disease. Of particular interest, the antibody response during active TB was directed against ∼0.5% of the total Mtb proteome and was highly enriched in extracellular and secreted proteins (Table1) [52]. In contrast, patients with either latent or treated TB demonstrated more heterogenous antibody reactivity and were enriched for reactivity to membrane-associated antigens. In a complementary study, the same group used protein microarrays to investigate IgG serum dynamics in a nonhuman primate model. In animals with active TB, the authors detected increased antibody responses against secreted Pro-Glu/Pro-Pro-Glu (PE/PPE) proteins, which have established roles in Mtb pathogenesis and immune modulation [53]. Similar results were obtained with human samples run in the same study, with a subset of 10 proteins more tightly correlated with advancing disease state. Notably, the integration of human and animal model data in this study highlights a strong correlation between IgG antibody specificity and disease outcome.

**Table 1:** IgG and IgA antibody targets identified in Mtb protein microarray studies

Rv number	Protein name	Study		
		Kunnath-Velayudhan *et al.* [52, 53]	Deng *et al.* [54]	Song *et al.* [55]
IgG antigen targets				
Rv3881c	ESX-1 secretion-associated protein EspB	x	x	
Rv3804c	Secreted Ag85A/mycolyltransferase	x		
Rv3874	Secreted antigen Cfp10/EsxB/MTSA-10	x		
Rv1860	Secreted glycoprotein 45–47 kDa antigen/MPT32	x		x
Rv1411c	Lipoprotein LprG	x		
Rv2031c	16 kDa antigen/alpha-crystallin	x		x
Rv0934	Glycolipoprotein 38 kDa antigen/PstS1	x		
Rv3616c	Conserved hypothetical protein	x		
Rv3864	Conserved hypothetical protein	x		
Rv1980c	Secreted antigen MPT64	x		
Rv0632c	Enoyl-CoA hydratase/isomerase superfamily	x		
Rv1984c	Secreted antigen Cfp21	x		
Rv2873	Surface lipoprotein antigen MPT83	x		
Rv1174c	Low molecular weight T-cell antigen/TB8.4	x		
Rv3763	Lipoprotein lpqH/19 kDa Antigen	x		
Rv0324	Sulfurtransferase		x	
Rv0537c	Probable integral membrane protein		x	
Rv1685c	Transcriptional regulator, TetR family		x	
Rv2072c	Precorrin-6Y C(5,15)-methyltransferase		x	
Rv3899c	Uncharacterized protein		x	
Rv1100	Conserved protein		x	
Rv1865c	Oxidoreductase, short-chain dehydrogenase/reductase family		x	
MT0124	Putative uncharacterized protein		x	
Rv2884	Probable transcriptional regulator protein		x	
MT3959	Putative uncharacterized protein		x	
Rv2564	Uncharacterized ABC transporter ATP-binding protein		x	
Rv1654	Acetylglutamate kinase		x	
Rv0440	60 kDa chaperonin 2		x	
Rv2853	PE-PGRS family protein PE_PGRS48			x
MT3033	Uncharacterized protein			x
Rv3810	Exported repetitive protein precursor			x
Rv0109	E-PGRS family protein PE_PGRS1			x
Rv0040c	Secreted proline-rich protein Mtc28			x
Rv3260c	Probable transcriptional regulatory protein WhiB-like WhiB2			x
Rv1748	Unknown protein			x
Rv3835	Conserved membrane protein			x
Rv0247c	Probable succinate dehydrogenase			x
Rv3405c	Possible transcriptional regulatory protein			x
Rv3822	Conserved hypothetical protein			x
Rv2770c	PPE family protein PPE44			x
Rv0652	50S ribosomal protein L7/L12 RplL			x
Rv0054	Single-strand binding protein Ssb			x
Rv3544c	Probable acyl-CoA dehydrogenase FadE28			x
Rv1731	Possible succinate-semialdehyde dehydrogenase			x
Rv0178	Probable conserved Mce associated membrane protein			x
Rv2306c	Uncharacterized protein Rv2306c			x
Rv2668	Possible exported alanine and valine rich protein			x
Rv3897c	Conserved hypothetical protein			x
Rv0983	Probable serine protease PepD			x
Rv0474	Probable transcriptional regulatory protein			x
Rv2831	Probable enoyl-CoA hydratase EchA16			x
Rv3912	Hypothetical alanine-rich protein			x
Rv1904	Conserved hypothetical protein			x
Rv2922c	Probable chromosome partition protein Smc			x
Rv2668	Possible exported alanine and valine rich protein			x
Rv0638	Probable preprotein translocase SecE1			x
Rv0831c	Conserved protein			x
Rv1651c	PE-PGRS family protein PE_PGRS30			x
IgA antigen targets				

Rv1411c	Conserved lipoprotein LprG			x
Rv1754c	Conserved protein			x
Rv0983	Probable serine protease PepD			x
Rv0052	Conserved protein			x
Rv2922c	Probable chromosome partition protein Smc			x
Rv0509	Probable glutamyl-tRNA reductase HemA			x
Rv1566c	Possible Inv protein			x

Subsequent to these initial studies, a second microarray platform was developed in which all Mtb proteins were expressed as GST-fusion proteins, allowing for protein purification prior to spotting onto microarray slides. By comparing antibody responses in active TB patients with patients that had undergone chemotherapy treatment, the authors identified 14 antigens that discriminate the two groups. Interestingly, only one of the identified antigens (ESX-1 secretion-associated protein EspB) overlapped with the antigens identified in the earlier microarray study (Table 1) indicating that Mtb antigens and ensuing antibody responses are also modulated following drug treatment [54]. A final study took advantage of the most recent advancement in Mtb proteome microarrays: the development of Nucleic Acid Programmable Protein Arrays (NAPPA) which allows for high throughput *in situ* expression of a large number of proteins using cell-free expression systems [56]. NAPPA arrays also offer improvements in protein folding, in the expression of membrane proteins, and in the inclusion of correct posttranslational modifications. In this study, the authors investigated alterations in antibody targets during HIV and Mtb coinfection, with the goal of developing point-of-care diagnostics for TB [55]. A total of 34 IgG and 8 IgA antigens were characterized as antibody targets during Mtb infection, either in the presence or absence of HIV coinfection. Antibody targets also differed between TB patients in the USA and South Africa, potentially due to the infecting Mtb strain or regional differences in disease state (e.g. later diagnosis of advanced disease in resource-limited settings with endemic disease).

When comparing the antibody targets identified in these four microarray studies, it is striking to note how little overlap exists across the patients with active TB disease (Table 1). The reasons for this are unclear but there are several possibilities: different infecting Mtb strains, different stages of disease in individual patients, differences in HLA-DR loci between individuals or technical effects of the distinct microarray platforms used [57, 58]. Toward this last point, the correct folding and posttranslational modifications of proteins may be a particular challenge for Mtb microarrays. For example, recombinant HBHA does not recapitulate the complex methylation patterns found on native HBHA, thereby rendering it unable to bind monoclonal HBHA-specific antibodies [59]. In summary, these microarray studies clearly demonstrate that the antibody response during natural infection with Mtb is dynamic, mirroring the changing availability of antigens along the spectrum of infection in humans, and differing with the status of coinfection or treatment with antibiotics. The observation that the majority of such antigens are found in the culture filtrate of liquid grown Mtb might indicate that these antigens are highly abundant during active disease in which the growth and/or spreading of granulomas is associated with increased Mtb replication and dissemination across the lungs. If true, the presence of such high antibody titers may be merely a result of high bacterial burden. On the other hand, the nature of antigens that elicit antibodies to prevent or contain Mtb infection during latent disease has not been adequately defined.

## THE ANTIBODY RESPONSE AFTER VACCINATION

Initial Mtb infection occurs with the inhalation of a small number of bacilli into the lung that are phagocytosed by alveolar macrophages and further enter the lung parenchyma. As the bacteria pass from infected macrophages to other cell types, either infected dendritic cells or inflammatory monocytes transport Mtb to the pulmonary lymph nodes, where adaptive T and B cell responses are initiated. Studies in mice have shown that the lag time between encounter with Mtb and the presence of adaptive immune responses is at least 2 weeks [4, 60]. Thus, a major goal in developing a new vaccine against Mtb is to reduce this delay. To date, BCG remains the only licensed TB vaccine, and it is administered at birth in many countries worldwide. Although BCG is poorly protective against pulmonary disease in adults, a recent trial has raised hopes that BCG revaccination of adolescents within a high risk, TB endemic setting can help prevent infection [61]. Mucosal or intravenous BCG vaccination in macaques was also recently shown to induce near sterilizing immunity to Mtb infection challenge, correlating with an increase in Mtb-specific IgG, IgA and IgM antibodies in the blood and bronchoalveolar lavage fluid (BAL) fluid [62–65]. IgG and IgM antibodies isolated from the BAL of BCG vaccination macaques have subsequently been shown to opsonize Mtb and enhance bacterial uptake by macrophages *in vitro* [64]. Most recently, antigen-specific IgM titers in BAL were shown to correlate with reduced bacterial burden following Mtb challenge although it is not yet clear to what extent this is directly responsible for protection *in vivo* [65]. Notably, MTBVAC—a live attenuated Mtb-derived vaccine that elicits more robust and rapid adaptive immune responses compared with BCG—is also capable of inducing opsonizing antibodies in macaques [64]. Although the antigenic targets of BCG are more limited compared with Mtb, vaccination in humans elicits several overlapping antibody targets, including antibodies against LAM [66, 67]. Interestingly, oral administration of BCG in a small subset of study participants led to increased anti-LAM IgA titers in tears, underscoring its capacity to induce mucosal antibody responses [66]. Another study on intradermal BCG vaccination of participants in the UK reported increased AM-specific IgG responses in the serum, which correlated with opsonization and killing of BCG by human macrophages [68]. Antibodies specific for antigen Ag85A were also detected in a case–control analysis of the MVA85A efficacy trial that aimed to test the safety and efficacy of the candidate TB vaccine MVA85A (Modified Vaccinia virus Ankara expressing Ag85A from *M. tuberculosis*) in infants. Here, elevated Ag85A-specific IgG in the serum associated with reduced risk of disease after BCG vaccination [69]. Finally, and similar to Mtb infection, BCG vaccination was shown to induce high avidity IgG antibodies to bulk surface antigens, and in one study of Japanese healthcare workers, tuberculosis glycolipid [70, 71].

## MUCOSAL ANTIBODY RESPONSES DURING MTB INFECTION

**Figure 1:**
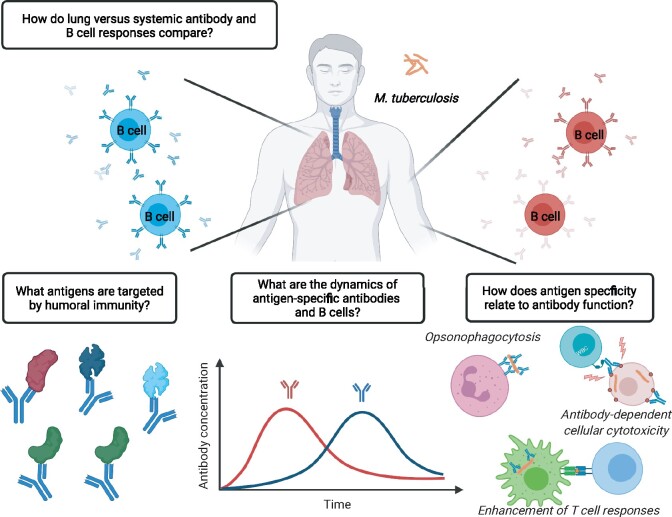
Open questions regarding the role of antibodies and B cells during Mtb infection. Created with BioRender (biorender.com/)

The majority of studies discussed so far have characterized antibody responses in blood after confirmed infection or high exposure to Mtb. Much less is known, however, about local antibody responses in the human lung, the primary site of Mtb infection. Only two studies have investigated this topic using a fraction of culture filtrate proteins, PstS1, alpha-crystallin or LAM to characterize antibody responses in human BAL during active disease. In the first study, increased levels of antigen-specific IgA and IgG were detected during active disease [72, 73]. In the second study, HBHA-specific memory B cells isolated from Mtb-exposed healthcare workers were biased toward IgA expression suggesting their mucosal origin. As noted earlier, depending on the isotype, monoclonal antibodies derived from these individuals differed in their ability to prevent epithelial cell infection *in vitro*, an effect which was also observed for polyclonal IgA and IgG antibodies isolated from serum [47]. Although this study highlights the potential importance of isotype in delineating antibody function, it is difficult to extrapolate these findings to other specificities or to an *in vivo* setting, particularly as the primary reservoir for Mtb infection is the alveolar macrophage compartment [74]. Given the paucity of studies on mucosal antibody responses during Mtb infection in humans, the specificity, dynamics, tissue compartmentalization and function of antibodies in the lung as compared the periphery represent significant knowledge gaps in the field (Fig. 1).

## B CELL ACCUMULATION IN THE LUNG CORRELATES WITH MTB PROTECTION

On the other hand, the recruitment and accumulation of B cells in the lung are clearly documented during Mtb infection. One of the hallmarks of Mtb infection is the generation of a lung granuloma, a highly organized clustering of immune cells that gather around a core of infected macrophages [75]. Individual granulomas can have marked differences in immune cell composition and bacterial control, likely reflecting the spectrum of Mtb disease. Notably, granulomas that contain clusters of B cells and T cell (known as inducible bronchus-associated lymphoid tissue or iBALT) are associated with protection from TB disease in human infection and animal models of disease [76, 77]. iBALT is induced by a variety of acute and chronic immune contexts (e.g. infection, asthma, pulmonary fibrosis and cancer) and is generally characterized by compartmentalized B and T cell areas, follicular dendritic cells, antigen-presenting cells, high endothelial venules, stromal cells and limited chemokine networks. Importantly, iBALT acts as a local hub for pulmonary immune responses by supporting productive germinal center (GC) reactions for the selection of high-affinity B cell effectors as well as the survival of memory B and long-lived plasma cells [78–80]. GC responses were also recently implicated in the development of protective antibodies targeting PstS1 from a patient with active TB [48]. Although monoclonal anti-PstS1 antibodies showed relatively moderate levels of somatic hypermutation, germline reversion of two antibodies dramatically reduced their ability to bind PstS1 as well as Mtb whole-cell lysate. In addition, iBALT can serve as a niche for the survival of long-lived Tfh cells, a specialized subset of CD4 T cells that support antibody production by B cells. In the case of influenza infection, sustained interaction between B cells and Tfh cells in the lung mucosa supports the ongoing selection of high-affinity effector and memory B cells [81]. Accordingly, late disruption of iBALT leads to a reduction in bone marrow plasma cells and systemic antibody titers. In keeping with these observations, inducible deletion of Bcl6, a key transcription factor for the differentiation of Tfh cells, impairs mucosal antibody secretion following pulmonary rechallenge [82, 83]. Mtb infection in mice also leads to the differentiation of lung-resident CD4 T cells with Tfh-like features [84]. Analogous to conventional Tfh differentiation in lymphoid organs, these PD1+KLRG1− CD4 T cells in the lung depend on intrinsic expression of Bcl6 and signaling through ICOS. Unlike KLRG1+ CD4 T cells, which more closely resemble inflammatory Th1 cells and are restricted to the lung vasculature, PD1+KLRG1− T cells reside in the lung parenchyma. Of particular interest, compared with lung KLRG+ CD4 T cells, PD1+KLRG1− CD4 T cells maintain higher expansion potential and confer superior host protection following transfer and Mtb challenge. These data are largely consistent with our recent observation that influenza-induced lung Tfh cells maintain higher expansion and differentiation plasticity compared with their Th1 counterparts [82]. Notably, CD4 T cells from a cohort of TB resisters—household contacts of active TB cases in Uganda—did not secrete IFN-γ but did upregulate the costimulatory molecule CD40L following *in vitro* stimulation [44]. CD40L is a surface marker typically expressed by Tfh effector cells, which is important for their ability to support B cell selection in GCs. These data suggest that active B cell–T cell collaboration helps to limit Mtb infection and is consistent with the detection of Mtb-specific antibodies in these individuals. Together these findings raise the possibility that ongoing interactions between B and T cells in the lung have a dual purpose: B cells support sustained expression of Bcl6 in Tfh cells, thereby stabilizing a reservoir of self-renewing memory T cells, while Tfh cells support ongoing B cell selection, survival and mucosal antibody secretion during chronic Mtb. Nevertheless, there is clearly more work to be done to determine if and how lung-localized antibodies and B cells contribute to surveillance and sterilizing immunity. Unraveling the mechanisms that promote and maintain tissue-resident B cells capable of rapid antibody production has important implications for the design of novel vaccines to combat TB.

## CONCLUSION

Over the last decade, increasing evidence suggests a protective role for antibodies and B cells during Mtb infection. A major limitation of most studies, however, is the small number of known Mtb antigens. A full understanding of antibody dynamics, function and anatomic compartmentalization requires a more thorough understanding of relevant antigenic targets during the course of natural infection and vaccination. Toward this end, microarray or comparable technologies will be instrumental. Once these targets are identified, it will be possible to test for specificities that might differentially drive host protective or pathologic outcomes. As the lung is the major route of Mtb infection and the predominant site of disease, further investigation of mucosal antibody selection, specificity and function is an essential area of research going forward. The clear correlation between iBALT formation and host protection during Mtb further highlights the potential of harnessing immune memory in the tissue by vaccination.
